# Clinical application of deep learning-based measurement of glomerular morphometry in IgAN

**DOI:** 10.3389/fimmu.2026.1910367

**Published:** 2026-08-04

**Authors:** Lin-lin Xu, Xing-kuo Zhang, Zhen-zheng Dong, Pei Chen, Lei Jiang, Li-jun Liu, Xu-jie Zhou, Ji-cheng Lv, Li Xiao, Su-fang Shi, Hong Zhang

**Affiliations:** 1 Renal Division, Peking University First Hospital; Kidney Genetics Center, Peking University Institute of Nephrology; Key Laboratory of Renal Disease, Ministry of Health of China; Key Laboratory of Chronic Kidney Disease Prevention and Treatment, Peking University, Ministry of Education, Beijing, China; 2 School of Artificial Intelligence, Beijing University of Posts and Telecommunications, Beijing, China

**Keywords:** computational pathology, deep learning, glomerular size, IgA nephropathy, renal pathology

## Abstract

**Background:**

Accurate glomerular pathology assessment is crucial for predicting outcomes and guiding treatment in IgA nephropathy (IgAN). Although the Oxford classification provides a standardized histological assessment, it is semi-quantitative and subjective. Identifying objective and interpretable renal pathological features could enhance prognostic prediction.

**Methods:**

We developed and validated a deep learning-based computational pathology pipeline for glomerular analysis in IgAN. A glomerular segmentation and classification model was developed based on deep convolutional neural networks in 183 periodic acid-Schiff (PAS)-stained whole slide images (WSIs) of renal pathology from IgAN patients, and was further applied to WSIs of 886 IgAN patients from a prospective cohort. Associations of glomerular morphometry with clinical phenotypes and prognosis were determined.

**Results:**

The models demonstrated glomerular segmentation performance with a Dice coefficient of 0.91 and classification performance with an AUC of 0.94. Through a comprehensive correlation analysis of quantitative morphometric characteristics and clinical parameters, we found that age, gender, and body mass index significantly influenced glomerular morphology. No association was found between proteinuria and glomerular size. Notably, a nonlinear relationship was observed between glomerular size and estimated glomerular filtration rate (eGFR), with patients having an eGFR between 30 and 60 ml/min/1.73m^2^ exhibiting larger glomerular size compared to those with eGFR<30 or eGFR≥60 ml/min/1.73m². A biphasic relationship between mean glomerular area and poor prognosis of IgAN was identified. The AUC of the prognosis prediction model increased from 0.79 to 0.84 when integrating glomerular histomorphometric into the International IgAN Prediction Tool.

**Conclusions:**

In conclusion, we developed an end-to-end pipeline for glomerular segmentation, classification, and morphometry in WSIs. These objective and highly reproducible quantitative characteristics of glomeruli may serve as a valuable tool for therapeutic guidance and prognostic assessment in IgAN.

## Introduction

Immunoglobulin A (IgA) nephropathy (IgAN), the most prevalent primary glomerulonephritis, whose clinical manifestations and pathological alterations are diverse (1–3).A recent study suggested that nearly all patients with IgAN will progress to kidney failure within lifetime (4). To prevent and delay the onset of kidney failure, early identification of those at risk for progression and early intervention are essential. Renal biopsy is currently considered the gold standard for diagnosing IgA nephropathy (5). The Oxford pathology classification is the most widely used internationally for IgA nephropathy, which can be used to predict the likelihood of disease progression. However, the reproducibility of the Oxford classification was not good. The VALIGA study discovered poor reproducibility between local and central pathologists. The primary reason is that although pathology is the most objective diagnostic method, the diagnostic conclusions drawn from it remain subjective. Therefore, it is crucial to identify objective and interpretable renal pathological features that can predict prognosis. Research has demonstrated that glomerular size could serve as a predictor for the progression of renal disease in patients with IgAN (6–8). However, early studies on glomerular morphometry relied on manual labeling, which was time-consuming and labor-intensive, making it less suitable for clinical application.

Recently, an increasing number of deep learning models have been utilized to extract morphological features of pathology (9–11) and facilitate automated analysis of kidney-related medical images, including chronic kidney disease prediction, predictive renal segmentation, automated diagnosis, and computer-aided disease classification (12–15). Hölscher et al. found that the smaller glomerular tuft areas were associated with worse prognosis in patients with IgAN (16). Previous studies had suggested that the maximal glomerular diameter was a prognostic indicator for disease progression in patients with IgAN (6, 7). Recently, Lucarelli et al. correlated deep learning-based automated reference renal histomorphometry with patient demographics and creatinine. They found that patients with lower SCr (presumably better renal function) tended to have smaller glomeruli (17).Therefore, it is essential to utilize AI for the automatic and precise measurement of glomerular morphology in a larger cohort of patients with IgAN. Additionally, conducting correlation analyses with the patients’ clinical phenotypes will help further investigate the dynamics of renal pathology and identify potential mechanisms.

In this study, we utilized a deep learning-based image analysis model to accurately segment and classify the glomeruli in WSIs of renal biopsies from patients with IgAN, followed by the automatic measurement of glomerular morphological features. These quantified features reflected morphological changes associated with different states of the disease and revealed associations of morphological alterations with clinical parameters and the disease progression in IgAN.

## Methods

### Study design and participants

**Figure 1:**
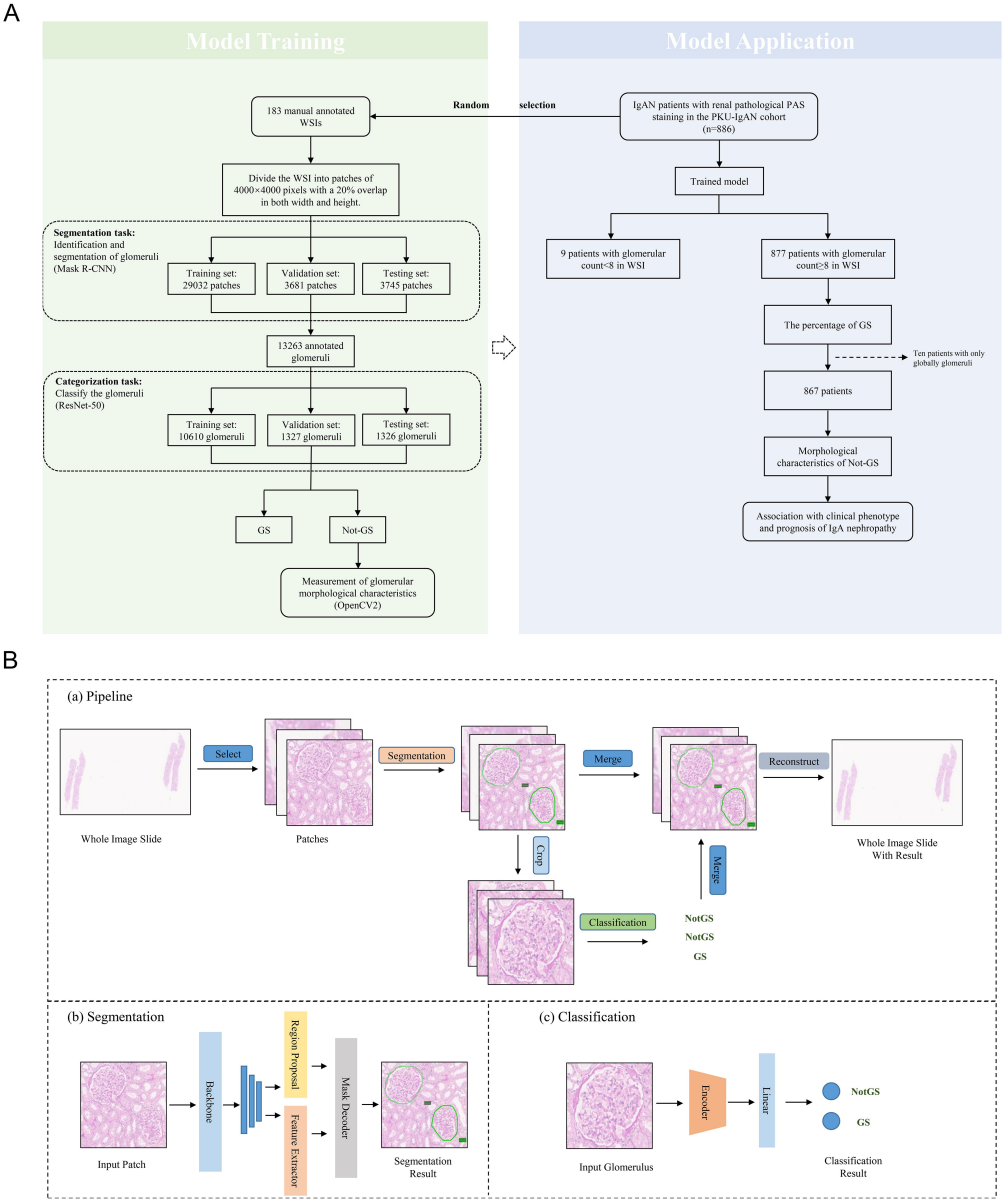
The flowchart of this study. **(A)** The entire workflow is demonstrated in this figure, including model training and model application. The training model consists of three components: a glomerular identification and segmentation model, a glomerular classification model, and a measurement for glomerular morphological features. The trained model was implemented in the IgA nephropathy cohort to evaluate the association between quantitative glomerular characteristics and clinical phenotypes. **(B)** The overall pipeline for the entire model development.

This study retrospectively examined IgAN patients from a cohort at Peking University first hospital, spanning the years 1997 to 2020. All patients with IgAN were diagnosed based on the histologic and immunofluorescence study of the renal biopsy. Patients who underwent regular follow-up at Peking University first hospital for at least one year and had complete clinical data at the time of biopsy were included in the analysis. The exclusion criteria were: (1) secondary IgAN (e.g., associated with Henoch-Schönlein purpura, lupus nephritis, chronic liver diseases, and other immunologic disorders); (2) patients without PAS-stained scanned sections were excluded; (3) poor WSI quality (large artefacts, insufficient scan quality, insufficient stain quality, and broken slides). Ultimately, 886 IgAN patients with clear PAS-stained WSIs were enrolled in this study. 183 of these patients were randomly selected for manual annotation of glomeruli. The boundaries of the entire glomeruli (glomerular capillaries, Bowman’s space, and Bowman’s capsule) were first manually outline, and then the glomeruli were classified as either globally sclerotic (defined as sclerosis involving the entire glomerular tuft, including obsolescent, solidified and disappearing global glomerulosclerosis) or nonglobally sclerotic by a trained medical postgraduate(Lin-Lin Xu) using Aperio ImageScope. Then, an expert nephropathologist (Su-Fang Shi)corrected all annotations. Any annotations of difficult or questionable structures were discussed in regular meetings with another expert nephropathologist(Lei Jiang). All annotators were required to adhere to a standard operating procedure. The final revised annotations served as ground truth for model training and evaluation. The above-trained model for glomerular segmentation and classification was utilized to predict the segmentation and classification of glomeruli in the WSIs of 886 IgAN patients. Then, the segmented glomeruli were subjected to feature extraction (**Figure 1A**).

This study was approved by the ethics committee of Peking University First Hospital [Ethical number: 2023yan225-001] and was conducted in accordance with the principles of the Helsinki Declaration. Written informed consent was provided by all participants.

### Segmentation model framework

To achieve precise glomerular localization and segmentation, we employed Mask R-CNN (14) as the foundational framework, enhanced with several key modifications tailored to whole-slide image (WSI) analysis. The model utilizes the Swin-Transformer (15) as the backbone network, leveraging its hierarchical feature extraction capabilities to capture multi-scale contextual information essential for accurate glomerular identification. The model consists of the following key components:

Region Proposal Network (RPN): The RPN generates candidate regions of interest within the image.

Mask Prediction Branch: The regions generated by the RPN are refined using a mask prediction branch to generate precise segmentation masks.

This architecture ensures effective feature extraction at multiple scales, crucial for handling the complexity and heterogeneity of renal tissue in WSIs. Given the high resolution of WSIs, we adopted a patch-based processing strategy to improve computational efficiency and scalability. To address the challenge of processing WSIs with large non-informative regions, we developed a novel dual-threshold filtering mechanism for effective patch selection in glomerular segmentation. This filtering strategy resulted in a curated dataset consisting of 36,458 high-quality patches of size 4000×4000, which were split into training (29,032), validation (3,681), and test (3,745) sets with an approximate ratio of 8:1:1. This curated dataset significantly reduced computational overhead while ensuring the model was trained on the most relevant tissue areas. Detailed procedure was described in the **Supplementary Methods**.

### Glomerular classification model training

For the classification of glomeruli, we utilized the ResNet50 architecture, a model renowned for its performance in image classification and object detection tasks. The ResNet50 backbone was used to extract high-level features, followed by the addition of a fully connected layer for final classification **Supplementary Figure 1**). To ensure accurate classification of glomeruli, we implemented a glomerulus-centric cropping strategy. The center points of each glomerulus were determined based on its annotated mask, and the image was cropped to 1000×1000 pixels. This crop size ensured that the entire glomerulus was captured, along with sufficient surrounding context. The dataset comprised 7,839 manually annotated glomeruli, including 1,808 globally sclerotic glomeruli (minority class) and 6,031 non-globally sclerotic glomeruli (majority class). To address this class imbalance, a multi-faceted strategy combining data-level and algorithm-level approaches was implemented. Further details were provided in **Supplementary Methods**.

Finally, a total of 13,263 annotated glomeruli were randomly allocated into training set (10,610), validation set (1,327), and test set (1,326) at a ratio of 8:1:1 for the development, validation, and testing of the glomerular classification model.

The overall pipeline for the entire model was shown in **Figure 1B**.

### Implementation details

For the glomerular segmentation model, Mask R-CNN with a Swin Transformer backbone was trained using a 1× schedule for 12 epochs. Stochastic gradient descent was used as the optimizer, with an initial learning rate of 0.02, momentum of 0.9, and weight decay of 0.0001. The learning rate followed a step decay schedule and was reduced by a factor of 10 at the 8th and 11th epochs. Linear warm-up was applied during the first 500 iterations, with a warm-up ratio of 0.001. The batch size was set to 16 images, corresponding to 8 GPUs with 2 images per GPU. Model checkpoints and validation evaluation were performed once per epoch. During training, data augmentation strategies, including random rotation, scaling, and elastic deformation, were applied to improve model robustness to variations in glomerular morphology and spatial arrangement.

For the glomerular classification model, a ResNet50 backbone initialized with ImageNet-pretrained weights was used, followed by a task-specific fully connected classification layer with two output nodes corresponding to globally sclerotic and non-globally sclerotic glomeruli. The model was trained using the AdamW optimizer with an initial learning rate of 1 × 10^-4^ and a weight decay of 1 × 10^-4^. The batch size was set to 16, and the model was trained for 50 epochs. A cosine annealing learning rate scheduler was adopted during training. To reduce the influence of class imbalance, globally sclerotic glomeruli were oversampled and augmented using random rotation within ±30°, horizontal and vertical flipping, and elastic deformation.

Based on the training, validation, and test sets described above, the training set was used to optimize model parameters, whereas the validation set was used to monitor model convergence and select the best-performing checkpoint. The independent test set was held out during model development and was used only for final performance evaluation.

### Measurement of glomerular morphological characteristics

We focused on the following interpretable glomerular morphological characteristics: area, diameter, perimeter, elongation, and eccentricity. These characteristics were calculated using the OpenCV2 toolkit based on the outputs of the localization and classification models. The formulas for some features were referenced from Hölscher et al (16. The details of the calculations were shown in **Supplementary Table 1**. The characteristics were calculated at the glomerular level, but were also summarized at the subject-level. The percentages of globally sclerotic glomeruli were extracted on WSI-level.

### Performance evaluation

The Dice coefficient, Jaccard index, average surface distance (ASD), precision, recall, and F1-score were used to evaluate the performance of the glomerular segmentation model. The Dice coefficient and Jaccard index are commonly used region-based metrics that measure the overlap between predicted segmentation masks and ground-truth annotations. The Dice coefficient ranges from 0 to 1, with higher values indicating better performance, and is mathematically equivalent to the pixel-level F1-score in binary segmentation tasks. The Jaccard index, also known as Intersection over Union, is defined as the ratio between the intersection and union of the predicted and ground-truth regions. ASD was used to evaluate boundary accuracy, with lower values indicating better alignment between predicted and ground-truth boundaries. Precision and recall were additionally reported to assess false-positive and false-negative segmentation errors. For the glomerular classification model, precision, recall, accuracy, F1-score, and area under the receiver operating characteristic curve (AUC) were used to evaluate classification performance.

### Statistical analysis

The sociodemographic and clinical variables were calculated and expressed as the mean ± standard deviation for variables with approximately symmetrical distributions, and median (interquartile range 25^th^-75^th^ percentile) for variables with skewed distributions. All categorical variables were expressed as frequencies and percentages. One PAS-stained WSI was available for each patient, and the glomerular characteristics of individual patients were assessed by averaging the area, diameter, perimeter, elongation, and eccentricity of all nonglobally sclerotic glomeruli for each patient. Spearman’s linear correlation analysis was conducted on the morphological features of glomeruli based on the patient-level that were manually annotated and those segmented by the same WSI using the model.

For association analysis of glomerular morphological characteristics and clinical characteristics, the Spearman correlation analysis was used for continuous clinical phenotypes. Statistical differences between groups were tested using the nonparametric Wilcoxon rank-sum test/Kruskal-Wallis test. For prognosis analysis, the primary endpoint was defined as ESKD or a ≥50% reduction in eGFR after diagnostic kidney biopsy. The second endpoint was defined as ESKD or a ≥30% reduction in eGFR after diagnostic kidney biopsy. Renal survival time from the composite endpoint event was calculated from the biopsy to the last follow-up. Follow-up time was censored if the patient was lost to follow-up. The renal survival analysis was conducted using the Kaplan-Meier method. Optimal cut-offs for all glomerular morphological characteristics were determined by computing the maximally selected log-rank statistic using the “survminer” R package. To investigate the potential nonlinear relationship between glomerular characteristics and risk of kidney progression event, we used the restricted cubic splines in Cox regression with three knots (“rms” R package).

Receiver operating characteristic (ROC) curve analysis, quantified by the area under the curve (AUC), was employed to compare the diagnostic performance of the International IgA Nephropathy Prediction Tool alone versus in combination with glomerular morphometric measurements for discriminating patients with adverse prognostic outcomes. The prediction score generated by the International IgA Nephropathy Prediction Tool was used as the baseline predictor. Three representative morphometric features, including mean glomerular area, mean eccentricity, and the percentage of globally sclerotic glomeruli, were selected because they reflect glomerular size, shape alteration, and chronic sclerotic burden, respectively. These variables were entered into a multivariable regression model. The regression coefficients were estimated from the data.

## Results

### Model performance

**Figure 2:**
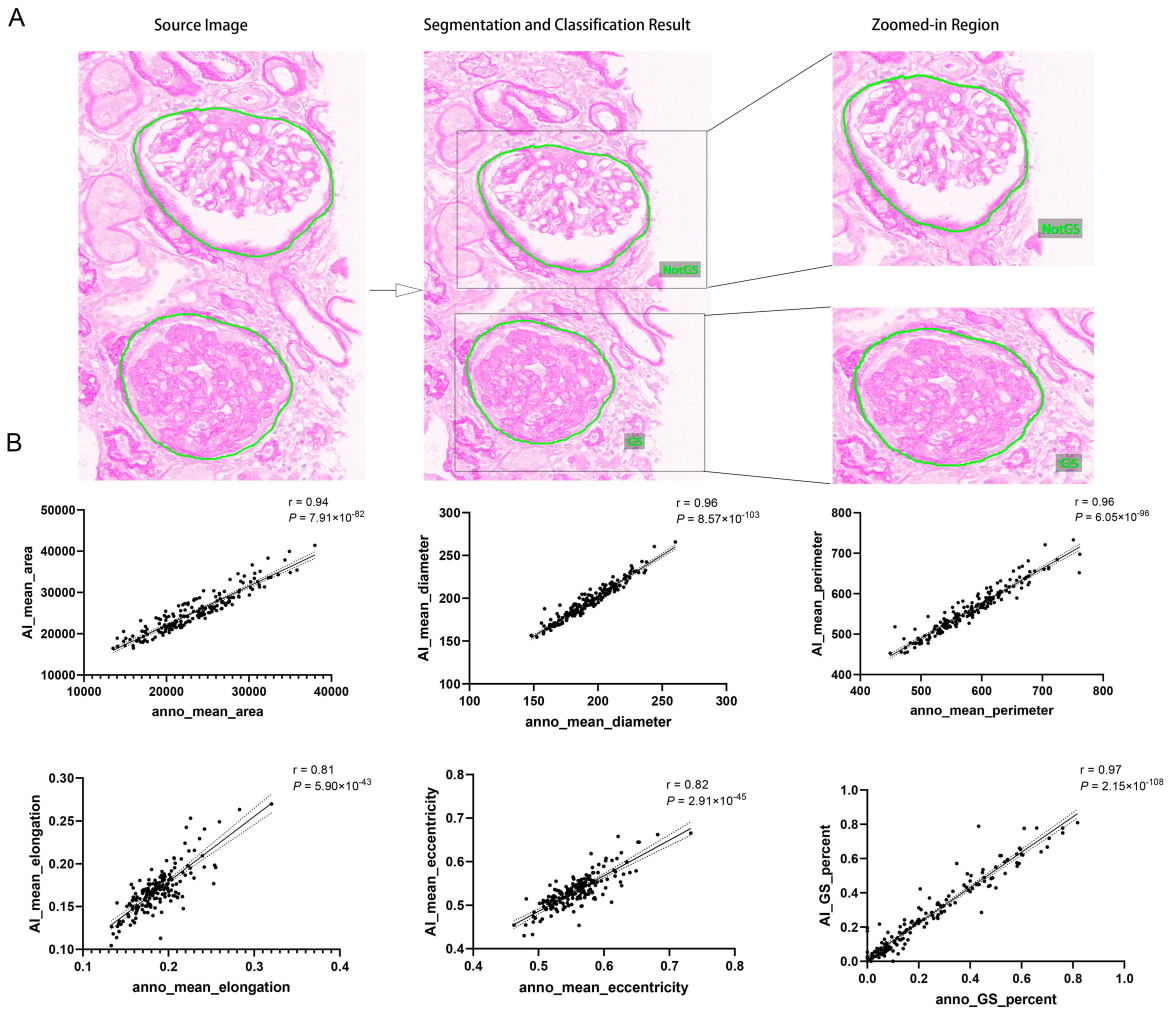
Assessment of glomerular segmentation, classification and morphometry model performance. **(A)** Representative visualization of glomerular segmentation and classification in periodic acid-Schiff (PAS)-stained kidney sections. The left panel shows the source image with glomerular boundaries delineated by the segmentation model. The middle panel shows the segmentation and classification results, with each glomerulus classified as globally sclerotic (GS) or non-globally sclerotic (NotGS). The right panel presents magnified views of the boxed regions in the middle panel. **(B)** Correlation analysis of mean quantitative features derived from manually annotated glomeruli versus deep learning-segmented glomeruli within the same whole-slide images (WSIs). Anno_mean_area, manually annotated glomeruli; AI_mean_area, deep learning-segmented glomeruli.

The ability of the Mask R-CNN to segment the glomeruli can be appreciated from **Figure 2A**. Our optimized model achieved strong performance in both segmentation and classification tasks. For segmentation, the model reached a Dice score of 0.91, corresponding to a pixel-level F1-score of 0.91, improving from the baseline Dice score of 0.80, and reduced ASD from 42.35 μm to 28.81 μm. For classification, the model attained a recall of 0.96, a precision of 0.97, an F1-score of 0.965, and an AUC of 0.94, improving from 0.87, 0.85, 0.86, and 0.84, respectively. These results indicate that our methodological refinements substantially enhanced model performance through systematic architectural and training process optimizations. Detailed results, including additional evaluation metrics, are provided in **Supplementary Tables 2**, **3**.

In addition, we correlated the morphological features of glomeruli measured from 183 WSIs based on manually labelled glomeruli with those measured from model segmentation-based glomeruli. As illustrated in **Figure 2B**, each feature measured from manually labeled glomeruli showed a very high correlation with its corresponding feature measured from model-segmented glomeruli, further demonstrating the reliability of the segmentation model.

### Association between glomerular morphometry and demography

A total of 886 WSIs from the PKU-IgAN cohort of patients with PAS-stained WSIs were applied to the already trained glomerular segmentation and classification model described above. Nine patients had a predicted glomerular count for WSI of less than 8. To ensure reliable and stable results, the data from these patients were not included in the analyses. Of the 877 patients with glomerular counts more than or equal to 8, 10 patients had only globally sclerotic glomeruli, so these 10 patients were also excluded. The final analysis included 867 patients assessing glomerular morphology and clinical phenotypes (**Figure 1A**). The specific clinical characteristics of these patients were shown in **Table 1**.

**Table 1:** The clinical characteristics of IgAN patients enrolled in this study.

Characteristics	All patients	Non-ESKD or eGFR decline≥50%	ESKD or eGFR decline≥50%	P value
number, (%)	867	772 (89.0%)	95 (11.0%)	
age, yr	37.13 ± 12.51	37.29 ± 12.64	35.83 ± 11.43	0.28
Male (%)	461 (53.2%)	397 (51.4%)	64 (67.4%)	3.00×10^-3^
BMI	24.85 ± 3.87	24.86 ± 3.89	24.75 ± 3.77	0.79
Systolic arterial pressure, mm Hg	126.56 ± 15.69	126.47 ± 15.67	127.32 ± 15.95	0.62
Diastolic arterial pressure, mm Hg	79.34 ± 10.76	79.22 ± 10.81	80.37 ± 10.33	0.33
Mean arterial pressure, mm Hg	95.08 ± 11.35	94.97 ± 11.35	96.02 ± 11.42	0.4
Proteinuria, g/d	1.13 (0.58-2.43)	1.02 (0.54-2.18)	2.55 (1.44-4.15)	2.60×10^-13^
eGFR, mL/min per 1.73 m^2^	74.33 ± 32.78	76.98 ± 32.05	52.79 ± 30.79	6.23×10^-12^
Microhematuria,/μL	50.05 (14.83-170.30)	51.90 (15.20-178.80)	40.90 (9.30-110.00)	0.09
Glomerular histomorphometric				
glomerular area, μm^2^	23096.41 (17290.65-27344.98)	23540.57 (19090.02-27509.76)	14494.80 (8704.36-26466.71)	2.00×10^-6^
glomerular diameter, μm	188.71 (163.86-205.60)	190.12 (170.89-205.77)	146.19 (113.76-200.33)	1.00×10^-6^
glomerular perimeter, μm	544.39 (475.60-593.86)	550.53 (496.96-595.57)	423.55 (330.49-573.37)	3.02×10^-7^
glomerular elongation	0.16 (0.14-0.18)	0.16 (0.14-0.18)	0.15 (0.13-0.17)	1.00×10^-3^
glomerular eccentricity	0.52 (0.49-0.55)	0.52 (0.50-0.55)	0.51 (0.47-0.54)	2.00×10^-3^
GS percent, %	14.46 (1.67-35.90)	13.98 (1.91-34.12)	31.15 (0-54.79)	5.00×10^-3^
Renal biopsy, n/n (%)				
Mesangial (M) 1	360/822 (43.8%)	306 (42.0%)	54 (58.1%)	3.00×10^-3^
Endocapillary (E) 1	251/822 (30.5%)	224 (30.7%)	27 (29.0%)	0.74
Glomerular sclerosis (S) 1	585/822 (71.2%)	502 (68.9%)	83 (89.2%)	4.40×10^-5^
Tubulointerstitial damage (T) T1+T2	262/822 (31.9%)	192 (26.3%)	70 (75.3%)	1.49×10^-24^
Crescent (C) C1+C2	538/822 (65.5%)	467 (64.1%)	71 (76.3%)	9.55×10^-8^

BMI, body mass index; eGFR, estimated glomerular filtration rate; ESKD, end-stage kidney disease.

**Figure 3:**
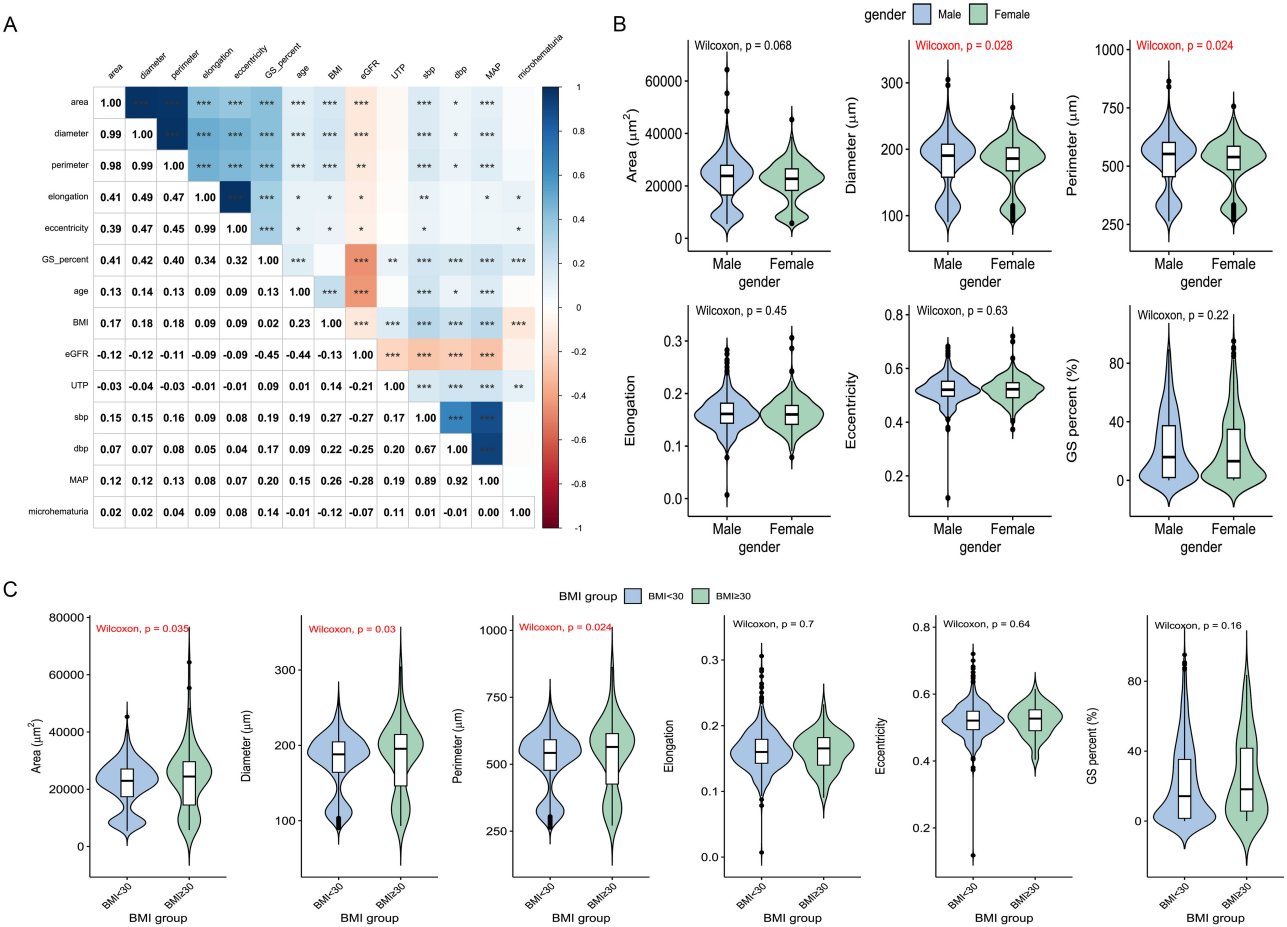
Association between glomerular morphometry and characteristics. **(A)** Heatmap showing the correlation between quantitative glomerular morphometric parameters and the clinical characteristics of the patients. The numbers within the heatmap represent Spearman’s correlation coefficient r, and the asterisk represents a significant correlation (*p < 0.05, **p < 0.01, ***p < 0.001). **(B)** Comparison of quantitative glomerular characteristics between male and female. **(C)** Comparison of quantitative glomerular characteristics between obese and non-obese patients. BMI, Body Mass Index. Differences between groups were tested using the Wilcoxon rank-sum test. The similar violin plots also reflect the high intrinsic correlations among these glomerular morphometric parameters (r = 0.98–0.99).

We first performed a correlation analysis between glomerular morphometric features. Undoubtedly, there was a significant correlation between glomerular area, diameter, and perimeter (**Figure 3A**). We also found a significant positive correlation between features that responded to glomerular size and those that responded to the degree of glomerular deformation; specifically, larger glomeruli tend to be less rounded. In addition, we found that the larger the glomerulus, the higher the percentage of sclerotic glomeruli (**Figure 3A**).

Glomerular size and degree of deformity were positively correlated with the patients’ age at the time of kidney biopsy, but the correlation coefficients were weak (Spearman’s r ranged from 0.09 to 0.13). The percentage of sclerotic glomeruli was positively correlated with age (r = 0.13, *P* = 7.60×10^-5^). The mean glomerular diameter (190.35μm (157.34μm -207.56μm) vs. 185.91μm (167.34μm -202.22μm), *P* = 0.03) and perimeter (552.18μm (454.69μm -602.28μm) vs. 539.13μm (484.11μm -585.43μm), *P* = 0.02) showed significant differences between males and females, while the mean glomerular area (23799.77μm (16457.51μm -27878.21μm) vs. 22711.02μm (18216.20μm -26552.05μm), *P* = 0.07) showed a tendency to differ between the two groups. Men tended to have a larger glomerular size than women (**Figure 3B**). Obese patients had larger glomeruli than non-obese patients (**Figure 3C**).

### Association between glomerular morphometry and clinical characteristics

**Figure 4:**
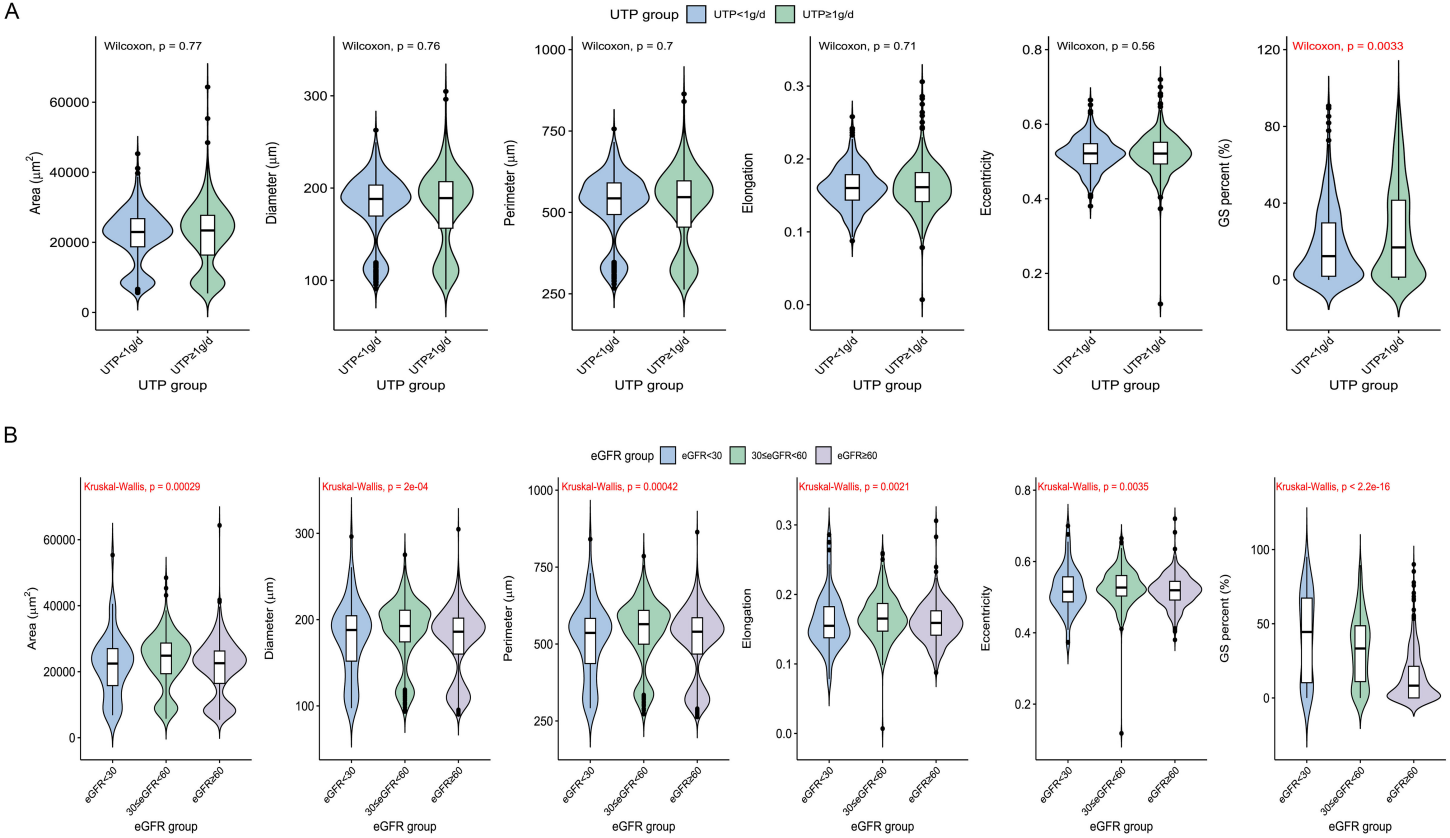
Association between glomerular morphometry and disease phenotype in IgA nephropathy. **(A)** Association of quantitative glomerular characteristics with the degree of proteinuria. **(B)** Differences in quantitative glomerular characteristics among eGFR subgroups. UTP, baseline proteinuria. eGFR, estimated glomerular filtration rate. Differences between groups were tested using the Wilcoxon rank-sum test. The similar violin plots also reflect the high intrinsic correlations among these glomerular morphometric parameters (r = 0.98–0.99).

As shown in **Figure 3A**, simple linear correlation analysis showed no correlation between glomerular size and proteinuria levels. Based on whether the proteinuria level was greater than 1 g/d, patients were grouped into high proteinuria and low proteinuria categories. There was no difference in glomerular size between these two groups. Proteinuria levels showed a significant positive correlation with the percentage of sclerotic glomeruli, which was elevated in patients with high proteinuria levels (**Figure 4A**). Organizing the patients according to their eGFR showed notable differences in glomerular size, the extent of glomerular deformation, and the percentage of sclerotic glomeruli among the groups. Interestingly, we observed that the mean glomerular area was larger in patients with eGFR of 30-60 [24858.59μm^2^ (19358.89μm^2^-28684.50μm^2^)] compared to those with an eGFR of < 30 [22462.53μm^2^ (15622.24μm^2^-27119.62μm^2^)] or ≥ 60 ml/min/1.73m^2^ [22567.81μm^2^ (16405.86μm^2^-26305.05μm^2^)]. Additionally, the mean glomerular elongation was also highest in patients with eGFR of 30–60 ml/min/1.73m^2^. The percentage of sclerosis glomeruli progressively increased with kidney function loss (eGFR<30 vs. 30≤eGFR<60 vs. eGFR≥60, 44.44% (10.09%-68.59%) vs. 33.33% [10.83%-48.71% vs. 8.27% (0-21.37%), *P* = 1.76×10^-34^] (**Figure 4B**).

**Figure 5:**
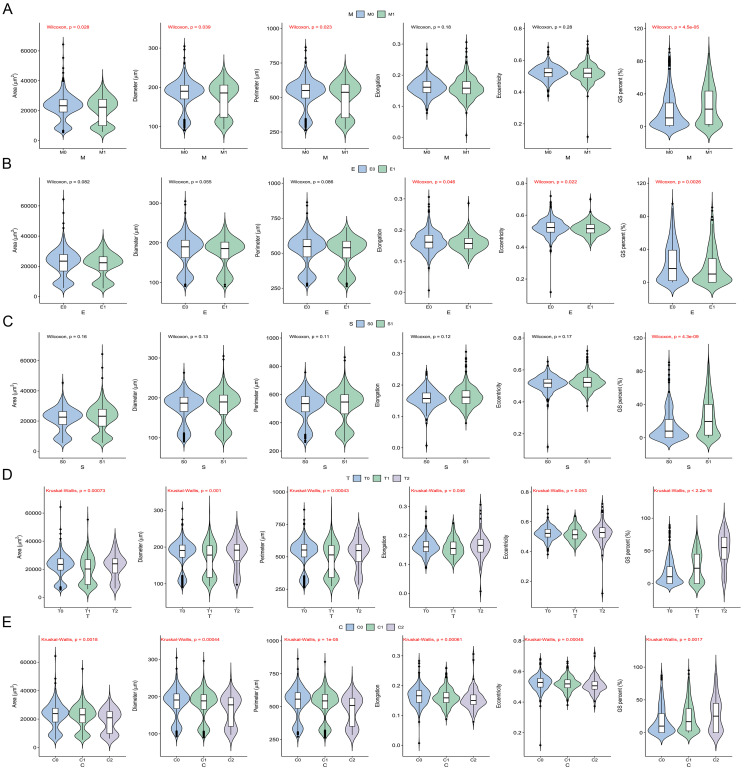
Association between glomerular morphometry and Oxford pathology scores in IgA nephropathy. **(A)** Association of quantitative glomerular characteristics with the M score in the Oxford pathology score. M0, M score=0; M1, M score=1 **(B)** Association of quantitative glomerular characteristics with the E score in the Oxford pathology score. E0, E score=0; E1, E score=1 **(C)** Association of quantitative glomerular characteristics with the S score in the Oxford pathology score. S0, S score=0; S1, S score=1 **(D)** Association of quantitative glomerular characteristics with the T score in the Oxford pathology score. T0, T score=0; T1, T score=1; T2, T score=2 **(E)** Association of quantitative glomerular characteristics with the C score in the Oxford pathology score. C0, C score=0; C1, C score=1; C2, C score=2. Differences between groups were tested using the Wilcoxon rank-sum test/Kruskal-Wallis test. The similar violin plots also reflect the high intrinsic correlations among these glomerular morphometric parameters (r = 0.98–0.99).

We also analyzed the relationship between the Oxford classification of IgA nephropathy and glomerular morphological features. Patients with M0 had a larger mean glomerular area than patients with M1 (23255.16μm^2^ (19007.38μm^2^-27284.13μm^2^) vs. 22291.25μm^2^ (9895.26μm^2^-27551.32μm^2^), *P* = 0.03). The percentage of sclerotic glomeruli was higher in patients with M1 than those with M0 (21.43% (2.71%-43.56%) vs. 10.69% (0.84%-29.03%), *P* = 4.50×10^-5^) (**Figure 5A**). The percentage of sclerotic glomeruli was lower in patients with E1 than those with E0 (10.00% (0-28.77%) vs. 16.67% (1.96%-38.89%), *P* = 3.00×10^-3^) (**Figure 5B**). The percentage of sclerotic glomeruli was higher in patients with S1 than those with S0 (19.61% (3.08%-40.00%) vs. 8.00% (0-21.98%), *P* = 4.28×10^-9^) (**Figure 5C**). The mean glomerular area was smaller in patients with T1 (20190.17μm^2^ (9157.91μm^2^-26873.46μm^2^)) compared to those with T0 (23503.35μm^2^ (19398.00μm^2^-27462.52μm^2^)) or T2(23898.25μm^2^ (16669.33μm^2^-27580.74μm^2^), *P* = 1.00×10^-3^). The higher the T score, the higher the percentage of sclerotic glomeruli (10.47% (0-25.90%) vs. 23.43% (0-45.00%) vs. 54.95% (36.91%-70.87%), *P* = 1.67×10^-25^) (**Figure 5D**). The higher the C score, the smaller the mean glomerular area was (23848.28μm^2^ (17764.93μm^2^-27671.98μm^2^) vs. 22985.84μm^2^ (17367.50μm^2^-27570.76μm^2^) vs. 20911.24μm^2^ (9726.59μm^2^-25391.40μm^2^), *P* = 2.00×10^-3^). The higher the C score, the higher the percentage of sclerotic glomeruli (10.00% (0-29.17%) vs. 16.77% (2.65%-36.81%) vs. 25.21% (0-45.20%), *P* = 2.00×10^-3^) (**Figure 5E**). The marked visual similarity among the violin plots is expected, as pairwise correlation analysis revealed extremely high correlations among area, maximal diameter, and perimeter (r = 0.98-0.99) and between eccentricity and elongation (r = 0.99), reflecting their intrinsic biological and mathematical interrelationships.

### Association between glomerular morphometry and prognosis

**Figure 6:**
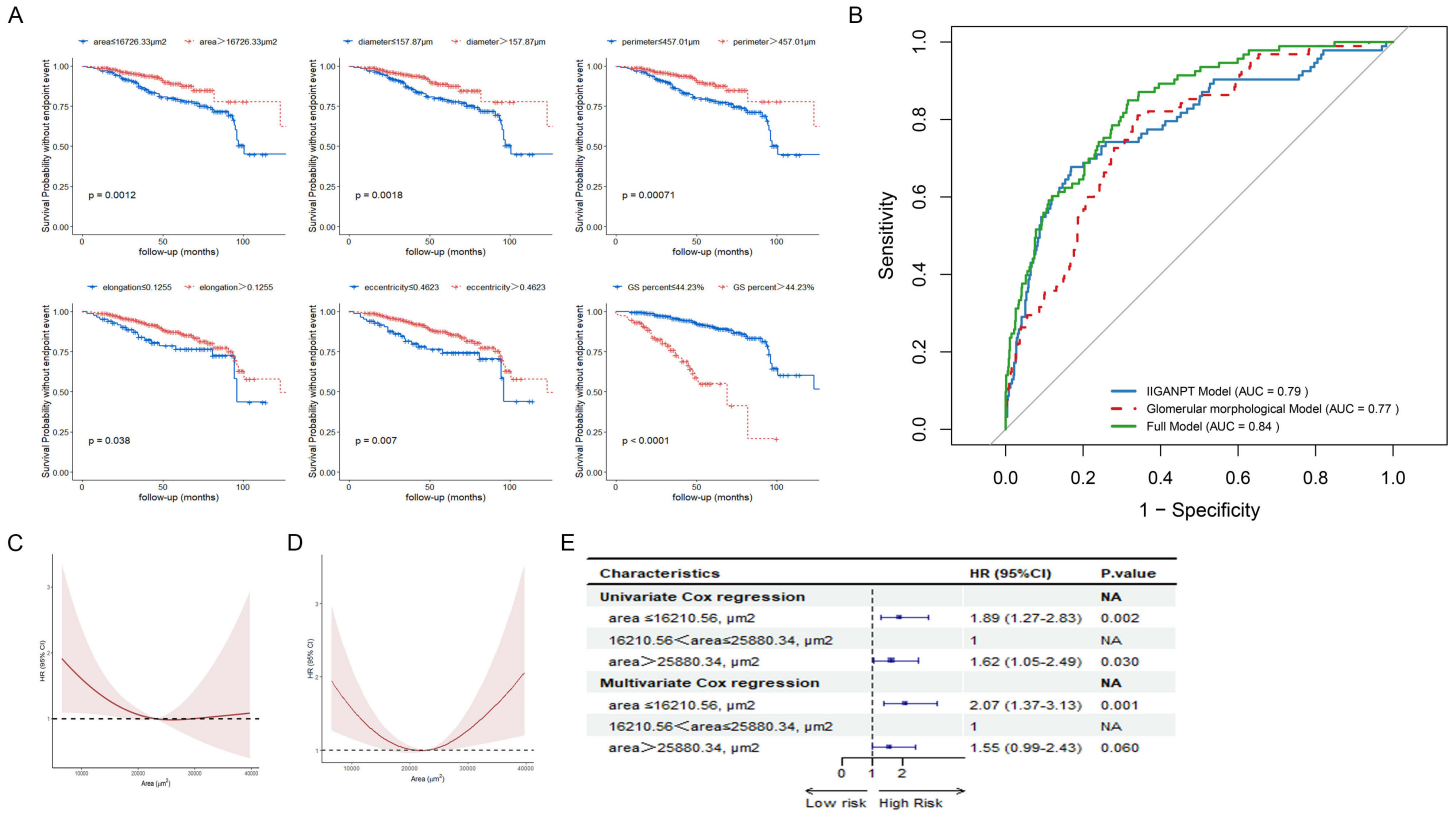
Quantitative glomerular characteristics are predictive of disease progression in IgA nephropathy. **(A)** Kaplan-Meier survival analysis based on quantitative glomerular characteristics. **(B)** Receiver operating characteristic curves and the AUC for models predicting the risk of the ESKD or eGFR decline ≥50% using the IIGANPT model with and without glomerular morphological model (the mean glomerular area, mean glomerular eccentricity, and the percentage of sclerotic glomeruli). **(C)** Association of mean glomerular area and adjusted HR of ESKD or eGFR decline ≥50% in patients with IgAN. Three knots were used to model restricted cubic splines. The estimated risk ratio is depicted by the solid line, while the 95% CI is represented by the shaded area. Models were adjusted for age, gender, and BMI. **(D)** Association of mean glomerular area and adjusted HR of the ESKD or eGFR decline ≥30% in patients with IgAN. Three knots were used to model restricted cubic splines. The estimated risk ratio is depicted by the solid line, while the 95% CI is represented by the shaded area. Models were adjusted for age, gender, and BMI. **(E)** Univariate and multivariate Cox regression analyses for the second endpoint (ESKD or eGFR decline ≥30%) across three categories of mean glomerular area defined by restricted cubic spline analysis. Multivariate models were adjusted for age, gender, and BMI.

During a median follow-up period of 36 months (23–56 months), 11.0% of patients (n = 95) reached the primary endpoint (ESKD or ≥50% decline in eGFR). We classified the glomerular morphological features based on the maximum choice log-rank statistic. In the univariate Cox regression analysis, patients with mean glomerular area of ≤16726.33μm^2^ had a 2-fold higher risk of reaching the primary endpoint compared to those with mean glomerular area of >16726.33μm^2^ (HR 2.01, 95% CI 1.31-3.08, *P* = 1.00×10^-3^). Similarly, the patients with mean glomerular eccentricity of ≤0.4623 had a 94% increased risk of the primary endpoint compared to those with mean glomerular eccentricity >0.4623 (HR 1.94, 95% CI 1.19-3.18, *P* = 8.00×10^-3^). Additionally, higher proportion of sclerotic glomeruli was strongly associated with an increased risk of kidney progression (HR 6.75, 95% CI 4.36-10.43, *P* = 8.61×10^-18^) (**Figure 6A**). After adjusting for age, gender, BMI, baseline proteinuria, eGFR, mean arterial pressure (MAP) and MEST-C at kidney biopsy, higher percentage of sclerotic glomeruli (HR 2.23, 95% CI 1.32-3.78, *P* = 3.00×10^-3^) remained significantly associated with a poorer prognosis. There was a trending correlation between smaller mean glomerular area (HR 1.55, 95% CI 0.97-2.46, *P* = 0.07) and smaller mean eccentricity (HR 1.72, 95% CI 1.00-2.95, *P* = 0.05) with poor prognosis (**Table 2**).

**Table 2:** The association between glomerular morphological characteristics and kidney prognosis in patients with IgAN.

Characteristics	Model 1			Model 2			Model 3		
	HR	95% CI	P	HR	95% CI	P	HR	95% CI	P
glomerular area ≤ 16726.33 μm^2^	2.01	1.31-3.08	1.00×10^-3^	1.69	1.06-2.68	0.03	1.55	0.97-2.46	0.07
glomerular diameter ≤ 157.87 μm	1.96	1.28-3.01	2.00×10^-3^	1.62	1.02-2.57	0.04	1.49	0.93-2.37	0.1
glomerular perimeter ≤ 457.01 μm	2.07	1.35-3.19	1.00×10^-3^	1.72	1.08-2.73	0.02	1.58	0.99-2.51	0.06
glomerular elongation ≤ 0.1255	1.71	1.02-2.84	0.04	1.22	0.71-2.10	0.47	1.48	0.85-2.58	0.16
glomerular eccentricity ≤ 0.4623	1.94	1.19-3.18	8.00×10^-3^	1.39	0.82-2.36	0.22	1.72	1.00-2.95	0.05
GS percentage ≥ 44.23%	6.75	4.36-10.43	8.61×10^-18^	3.24	1.96-5.36	5.00×10^-6^	2.23	1.32-3.78	3.00×10^-3^

Model 1: unadjusted; Model 2: Adjusted for age, gender, BMI, baseline proteinuria, eGFR, and MAP at kidney biopsy. Model 3: Model 2 and MEST-C. The composite kidney endpoint was ESKD or ≥50% decline in eGFR.

To clarify the prognostic value of glomerular morphological features in patients with IgAN, incorporating these glomerular morphological features (The mean glomerular area, mean eccentricity, and the percentage of sclerotic glomeruli) into the International IgA Nephropathy Prediction Tool without race (IIGANPT) (18) model enhanced the prediction of renal outcomes compared to using the IIGANPT model alone. Specifically, the addition of these features increased the area under the curve (AUC) from 0.79 for the IIGANPT model to 0.84, significantly improving the model’s ability to predict renal outcomes after biopsy, as illustrated in **Figure 6B**.

Given the non-linear relationship between glomerular size and patients’ baseline eGFR noted in the results above, we further analyzed whether there was a non-linear relationship between glomerular morphological characteristics and prognosis for all patients using the restricted cubic spline analysis with three knots. This analysis was adjusted for age, gender, and BMI. In the restricted cubic spline analysis, the number of knots is typically chosen to be between 3 and 5. Using too few knots (e.g., 2) may lead to underfitting, while using too many (more than 5) may result in overfitting. Considering our sample size, we decided to use three knots. The mean glomerular area exhibited a *U*-shaped trend concerning the risk of developing ESKD or a ≥50% reduction in eGFR (**Figure 6C**). A similar *U*-shaped trend was also observed for mean glomerular diameter, perimeter, elongation, and eccentricity (**Supplementary Figure 2**).

In sensitivity analyses using a more inclusive composite endpoint (ESKD or a ≥30% reduction in eGFR), which occurred in 18.3% of patients (n = 159) during follow-up, similar patterns emerged. According to univariate Cox regression analysis, patients with mean glomerular area of ≤16726.33μm^2^ had a 60% increased risk of reaching the second endpoint compared to those with mean glomerular area of >16726.33μm^2^ (HR 1.60, 95% CI 1.15-2.23, *P* = 5.00×10^-3^). The patients with mean glomerular eccentricity ≤0.4623 had a 70% increased risk of the second endpoint compared to those with mean glomerular eccentricity of >0.4623 (HR 1.70, 95% CI 1.14-2.53, *P* = 9.00×10^-3^). Furthermore, a high percentage of sclerotic glomeruli was associated with the increased risk of kidney progression events (HR 4.48, 95% CI 3.12-6.41, *P* = 2.99×10^-16^) (**Supplementary Figure 3**; **Supplementary Table 4**). We conducted additional subgroup analyses to categorize patients into three groups based on eGFR levels: eGFR<30, 30≤eGFR<60, and eGFR≥60 ml/min/1.73m^2^. Similarly, as the percentage of sclerotic glomeruli increased, the prognosis worsened, irrespective of the group. We also discovered that smaller glomerulus size was associated with poorer prognosis, whether the eGFR was <30 (**Supplementary Figure 4**) or eGFR≥60 ml/min/1.73m^2^ (**Supplementary Figure 5**). However, no association between glomerular size and prognosis was observed in the 30≤eGFR<60 group (**Supplementary Figure 6**). In the analysis using restricted cubic spline, a *U*-shaped relationship was identified between mean glomerular area and the second endpoint (**Figure 6D**). A similar *U*-shaped relationship was also observed between mean glomerular diameter, perimeter, elongation, and eccentricity with respect to the second endpoint (**Supplementary Figure 7**). When evaluating the mean glomerular area, we found that patients with a mean glomerular area of ≤16210.56 μm^2^ had an increased risk of poor prognosis (HR 1.89, 95% CI 1.27-2.83, *P* = 2.00×10^-3^). Similarly, those with a mean glomerular area >25880.34 μm^2^ also faced a heightened risk in comparison to patients whose mean glomerular area was between 16210.56 μm^2^ and 25880.34 μm^2^ (HR 1.62, 95% CI 1.05-2.49, *P* = 0.03) (**Figure 6E**). Additionally, to ensure the reliability of the results, we have similarly analyzed the results based on four nodes (**Supplementary Figure 8**) and five nodes (**Supplementary Figure 9**).

## Discussion

In this study, we employed deep learning to perform morphological measurements of nonglobally sclerotic glomeruli in IgA nephropathy and analyzed their correlation with renal prognosis. Our findings reveal that glomerular morphology in IgA nephropathy serves as an independent risk factor for renal prognosis. However, unlike previous studies, this risk is curvilinear, with both enlarged and reduced glomeruli being indicative of poor renal prognosis. Glomerular morphology is an important pathological supplement to the Oxford classification of IgA nephropathy. The renal prognostic ability of the International IgAN Prediction Tool was significantly improved by adding the glomerular features (the mean glomerular area, mean glomerular eccentricity, and the percentage of sclerotic glomeruli).

Recent studies have demonstrated the expanding application of artificial intelligence in healthcare (19–26). These studies further support the potential of deep learning in medical image analysis and clinical decision support, while highlighting the need for disease-specific model development and rigorous clinical validation. Renal pathology serves as a crucial reference for diagnosing, treating, and evaluating the prognosis of IgA nephropathy. The renal pathology associated with IgA nephropathy is diverse (27). While the Oxford MEST-C score has been used to guide clinical practice, several studies have demonstrated its limited effectiveness in treatment guidance and prognosis assessment. Furthermore, there is often low consistency in MEST-C scores between different centers (28, 29). This highlights the need to explore other simple and reliable quantitative indicators to provide better clinical guidance. Glomerular size is an easily measurable parameter. Larger glomeruli have been shown to play a crucial role in both animal experimental models and human outcomes in renal disease, and they may serve as predictors of long-term kidney function (30). Glomerular size is a classical and objective quantitative indicator, and due to its simplicity and clinical relevance, it is considered an adequate and desirable measure for routine clinical applications. In the past, the need for manual measurement made this process time-consuming and labor-intensive, preventing it from reaching its full potential in clinical settings. The application of artificial intelligence may enhance the clinical use of glomerular size for selecting the therapeutic strategies and assessing prognosis in IgA nephropathy.

To study the morphological characteristics of glomeruli, accurate segmentation of glomeruli is essential for extracting their morphological features. Globally sclerotic glomeruli have lost their normal physiological function, and their presence can obscure the identification of large glomeruli when assessing the average size of glomeruli (6, 31). Therefore, it is essential to measure only non-globally sclerotic glomeruli when calculating the mean morphological characteristics of glomeruli at the individual level. Further, we utilized OpenCV2 to automatically measure the morphological features of non-globally sclerotic glomeruli. Due to its straightforward application in clinical routines, we based our examination on the boundary of Bowman’s capsule as a morphometric parameter of the glomerulus. While a significant amount of glomerular morphology data can be obtained, these measurements are only meaningful if they have biological or clinical relevance. Therefore, we correlated glomerular morphologic features and the proportion of globally sclerotic glomeruli with the demographic characteristics, clinical phenotypes, and prognosis of patients with IgAN. There was a very strong correlation among mean glomerular area, mean maximum diameter, and mean perimeter. Since the mean glomerular area contains more information, subsequent analyses and discussions have focused on it. The determinants of glomerular size in IgAN include hyperfiltration, which occurs due to factors like reduced nephron numbers and body mass index (BMI). This hyperfiltration can lead to glomerular enlargement. On the other hand, disease-related glomerular injury, characterized by inflammation, proliferation, and sclerosis, may lead to glomerular shrinkage. The findings of this study support these concepts. We also observed a nonlinear relationship between the mean glomerular area and eGFR. Patients with eGFR<30 and those with eGFR≥60 ml/min/1.73m^2^ had comparable mean glomerular areas, whereas patients with eGFR between 30 and 60 ml/min/1.73m^2^ exhibited significantly larger glomeruli. Biologically, this pattern is consistent with a possible three-phase trajectory: (i) in earlier stages, surviving nephrons undergo compensatory hypertrophy in response to hyperfiltration; (ii) during intermediate stages (eGFR 30–60 ml/min/1.73m^2^), glomerular hypertrophy is most pronounced, reflecting sustained hemodynamic overload; and (iii) in advanced disease, hypertrophied glomeruli ultimately lose their compensatory capacity, collapse, and scar, resulting in smaller or sclerotic glomeruli. This finding was consistent with previous studies and hypotheses. Some injured glomeruli displayed hypertrophy as an early adaptive response before progressing to sclerosis and collapse, indicating that glomerular hypertrophy precedes the onset of glomerulosclerosis. This may reflect a pathological response of the glomerulus to alterations in hemodynamics or a compensatory circulatory mechanism, which was supported by both animal and human studies on renal disease (32–35). Furthermore, research on IgA nephropathy has shown that normal and sclerotic glomeruli exhibited similar sizes (6). In addition, as renal function declined, the remaining unaffected glomeruli may hypertrophy to compensate for the lost filtration capacity (36). However, as renal dysfunction progresses, these hypertrophied glomeruli eventually lose compensation and atrophy and become sclerosis. Taken together, these mechanisms suggest that the relationship between glomerular size and kidney function is inherently nonlinear, and that both glomerular enlargement and shrinkage may carry distinct biological and clinical implications.

Beyond the nonlinear relationship between glomerular size and eGFR, we further identified a biphasic (*U*-shaped) association between mean glomerular area and renal outcomes, in which both enlarged and reduced glomeruli were associated with a higher risk of disease progression. This pattern may reflect two distinct biological states: (i) markedly enlarged glomeruli representing an ongoing hyperfiltration and maladaptive remodeling phase, in which patients are at high risk of accelerated damage despite seemingly preserved nephron mass; and (ii) abnormally small glomeruli indicating advanced structural loss and reduced nephron reserve, in which little potential for recovery remains. From a clinical perspective, these findings suggest that mean glomerular area should not be interpreted in a purely linear fashion-”larger is better” or “smaller is better”-but rather in relation to disease stage. Patients with either markedly enlarged or markedly reduced glomeruli may warrant closer monitoring and more intensive risk management, even when traditional clinical parameters appear similar. Future studies are needed to determine whether integrating quantitative glomerular morphometry into existing prognostic models can improve risk stratification and guide individualized treatment in IgAN.

There are some limitations that should also be noted. First, the current study was conducted at a single center, which may limit its generalizability. External validation in an independent, multicenter dataset is essential to confirm the model’s robustness across different populations and staining variations. Future work will prioritize multicenter collaborations to prospectively validate the clinical utility of our model in real-world practice. Second, this study primarily developed a glomerular segmentation model, excluding tubular segmentation and the measurement of related parameters. Further research will focus on analyzing the relationship between tubular morphological features and clinical phenotype. Third, because this is a retrospective study, potential confounding factors such as variations in treatment strategies could not be fully controlled. It will be necessary to conduct prospective studies to validate our findings and facilitate their routine clinical implementation. Fourth, the morphological assessment in this study was primarily based on PAS staining. Further work on other stained sections should be carried out to facilitate the application of these findings in a broader range of centers where PAS staining is not available. Finally, larger sample sizes of measured glomerular morphological characteristics in both patients with IgAN and healthy individuals are needed to establish appropriate thresholds for predicting poor prognosis.

In conclusion, our study utilized a larger sample size and systematically analyzed the glomerular morphometric characteristics in relation to the clinical phenotype and prognosis of IgAN, which offered insights into the dynamics of glomerular changes during disease progression and the underlying mechanism, providing a rationale for the use of subsequent treatments.

## Data Availability

The raw data supporting the conclusions of this article will be made available by the authors, without undue reservation.
