# Supplementary material for: Clinical application of deep learning-based measurement of glomerular morphometry in IgAN
Source: Front Immunol. 2026 Aug 4;17:1910367. doi: 10.3389/fimmu.2026.1910367 (PMC13481774; doi:10.3389/fimmu.2026.1910367)
Supplement: Supplementary file 1 [file Table1.docx]

**Supplemental Material**

**Table of Contents for the supplemental material**

**Supplemental Methods**

**Supplemental Figure 1. The architecture of the ResNet50 training framework.** The framework comprises data preprocessing, a ResNet50-based backbone for feature extraction, and a head module for classification. In the data preprocessing stage, glomeruli of 1000x1000 size are extracted from the source image, followed by oversampling to balance the dataset. Data augmentation techniques are then applied to enhance model generalization. The backbone network adopts ResNet50, where the input image is first processed through an initial layer before passing through multiple residual blocks. Each ResBlock consists of a downsampling operation and residual connections to facilitate gradient flow and improve feature learning. After passing through three stacked residual blocks, the extracted features are forwarded to the head module, where a multilayer perceptron (MLP) processes them for classification. The final classification output is supervised by a hybrid loss function, comprising $\mathcal{L}_{local}$ and $\mathcal{L}_{weighted}$, which are computed based on the ground-truth labels. These loss functions help to refine the model's ability to distinguish between globally sclerotic glomeruli (GS) and non-GS glomeruli, ensuring accurate classification and robust model performance.

**Supplemental Figure 2.** **Non-linear association between quantitative glomerular characteristics and ESKD or ≥50% reduction in eGFR.** Association of quantitative glomerular morphological characteristics and adjusted HR of 50% eGFR decline or ESKD in patients with IgAN. Three knots were used to model restricted cubic splines. The estimated risk ratio is depicted by the solid line, while the 95% CI is represented by the shaded area. Models were adjusted for age, gender, and BMI.

**Supplemental Figure 3.** **The association between glomerular morphological characteristics and ESKD or ≥30% reduction in eGFR.**

**Supplemental Figure 4.** **Kaplan-Meier survival curves based on quantitative glomerular characteristics in patients with eGFR <30 ml/min/1.73m^2^.** Optimal cut-offs for all glomerular morphological characteristics were determined by computing the maximally selected log-rank statistic using the “survminer” R package.

**Supplemental Figure 5. Kaplan-Meier survival curves based on quantitative glomerular characteristics in patients with eGFR ≥60 ml/min/1.73m^2^.** Optimal cut-offs for all glomerular morphological characteristics were determined by computing the maximally selected log-rank statistic using the “survminer” R package.

**Supplemental Figure 6. Kaplan-Meier survival curves based on quantitative glomerular characteristics in patients with 30≤eGFR <60 ml/min/1.73m^2^.** Optimal cut-offs for all glomerular morphological characteristics were determined by computing the maximally selected log-rank statistic using the “survminer” R package.

**Supplemental Figure 7.** **Non-linear association between quantitative glomerular characteristics and ESKD or ≥30% reduction in eGFR.** Association of quantitative glomerular morphological characteristics and adjusted HR of 30% eGFR decline or ESKD in patients with IgAN. Three knots were used to model restricted cubic splines. The estimated risk ratio is depicted by the solid line, while the 95% CI is represented by the shaded area. Models were adjusted for age, gender, and BMI.

**Supplemental Figure 8.** **Non-linear association between quantitative glomerular characteristics and ESKD or ≥30% reduction in eGFR based on four knots.** Association of quantitative glomerular morphological characteristics and adjusted HR of 30% eGFR decline or ESKD in patients with IgAN. Four knots were used to model restricted cubic splines. The estimated risk ratio is depicted by the solid line, while the 95% CI is represented by the shaded area. Models were adjusted for age, gender, and BMI.

**Supplemental Figure 9.** **Non-linear association between quantitative glomerular characteristics and ESKD or ≥30% reduction in eGFR based on five knots.** Association of quantitative glomerular morphological characteristics and adjusted HR of 30% eGFR decline or ESKD in patients with IgAN. Five knots were used to model restricted cubic splines. The estimated risk ratio is depicted by the solid line, while the 95% CI is represented by the shaded area. Models were adjusted for age, gender, and BMI.

**Supplemental Figure 10.** **The architecture of the Mask R-CNN training framework.** The framework consists of multiple stages, including data preprocessing, backbone feature extraction, region proposal generation, fine-tuning, and post-processing. In the data preprocessing stage, the source image is cropped into 4000x4000 patches with a 20% overlap rate, followed by a dual-threshold filtering mechanism to refine the patches before feeding them into the model. The backbone utilizes a Swin Transformer to extract hierarchical feature maps, which are then processed by the neck module. The neck consists of a Region Proposal Network (RPN) that generates candidate regions of interest (ROIs), further refined through a convolutional neural network (CNN) to improve proposal quality. In the head module, the selected ROIs are passed through a multilayer perceptron (MLP) to generate mask predictions. The model is optimized using a hybrid loss function consisting of $\mathcal{L}_{region}$ and $\mathcal{L}_{boundary}$, which enhance segmentation accuracy by leveraging both regional and boundary information. To further improve performance, fine-tuning strategies such as a cosine annealing learning rate scheduler and data augmentation techniques are applied during training. Finally, in the post-processing stage, the predicted masks undergo refinement using a non-maximum suppression algorithm to eliminate redundant detections and enhance final segmentation quality.

**Supplemental Table 1.** Calculation of morphological characteristics of glomeruli.

**Supplemental Table 2.**  **Ablation study of optimization strategies for glomerular segmentation model**

**Supplemental Table 3.** Ablation study of optimization strategies for glomerular classification model

**Supplemental Table 4.** The association between glomerular morphological characteristics and second endpoint in patients with IgAN

**Supplemental Table 5.** Benchmarking results of candidate models across segmentation and classification metrics

**Supplemental Methods**

**Base Model Selection**

The model selection was validated through preliminary experiments conducted on carefully constructed subsets:

For glomerular segmentation: Training subset 5,000 patches (randomly sampled from final training set of 29,032 patches); Validation subset: 1,000 patches (representing 27% of final validation set of 3,681 patches); Testing subset: 1,000 patches (27% of final test set of 3,747 patches). Mask R-CNN achieved superior segmentation performance (Dice=0.80) compared to alternative architectures (0.76-0.78).

For glomerular classification: Training subset: 2,000 glomerular images (19% of final training set of 10,610 glomeruli); Validation subset: 500 glomeruli (38% of final validation set of 1,327 glomeruli); Testing subset: 500 glomeruli (38% of final test set of 1,326 glomeruli). ResNet50 demonstrated better classification performance (AUC=0.84) versus other models (0.79-0.83).

Complete comparative results across all evaluation metrics are presented in **Supplemental Table 4**. Ultimately, Mask R-CNN and ResNet50 were selected as our baseline models for their demonstrated performance advantages.

**Patch-based Processing**

Given the high resolution of WSIs, we adopted a patch-based processing strategy to improve computational efficiency and scalability. Each WSI was divided into overlapping patches of 4000×4000 pixels, with a 20% overlap in both the horizontal and vertical dimensions. This overlap ensures comprehensive coverage of glomerular structures while minimizing edge artifacts.

However, the patch-based approach introduces the challenge of redundant or overlapping segmentation results when the patches are reassembled into the original WSI coordinate system. To resolve this issue, we implemented a Non-Maximum Suppression (NMS) algorithm. The algorithm calculated the Intersection over Union (IoU) for each pair of overlapping segmentation results and retained the prediction with the highest confidence score while suppressing redundant or lower-confidence detections. This strategy efficiently handled high-resolution data, ensuring both computational efficiency and segmentation accuracy.

**Data Preprocessing**

To address the challenge of processing WSIs with large non-informative regions, we developed a novel dual-threshold filtering mechanism for effective patch selection in glomerular segmentation. This mechanism combined color intensity and texture complexity metrics:

Color Intensity: Only patches with mean RGB values below 230 were retained, ensuring sufficient tissue darkness.

Texture Complexity: Patches with a standard deviation greater than 10 were selected, ensuring that biologically relevant tissue areas with sufficient structural complexity were preserved.

**Training Strategy for Segmenting Adjacent Glomeruli**

Accurately segmenting densely clustered glomeruli is challenging due to their similar visual characteristics and close spatial proximity. To address this, we proposed a two-pronged training strategy. A dataset enriched with instances of closely adjacent glomeruli was curated. This ensured that the model was exposed to the most challenging cases, enhancing its ability to segment densely packed glomeruli in real-world scenarios. An adaptive loss function was introduced to improve segmentation accuracy, particularly in distinguishing the boundaries between adjacent glomeruli. The loss function consists of two components:

Region Segmentation Loss: A weighted cross-entropy loss that prioritizes the accurate segmentation of glomerular regions.

$$\mathcal{L}_{\text{region}}=-\frac{1}{N}\sum_{i=1}^{N} \left[ w_{\text{pos}}\cdot y_{i}log(p_{i})+w_{\text{neg}}\cdot(1-y_{i})log(1-p_{i}) \right]$$

where $N$ is the total number of pixels,$y_{i}$ is the ground truth label (1 for glomerulus, 0 for background), $p_{i}$ is the predicted probability of pixel $i$ belonging to a glomerulus, $w_{\text{pos}}$ and $w_{\text{neg}}$ are weights to address class imbalance, with $w_{\text{pos}}$ being higher to emphasize the importance of correctly identifying glomeruli.

Boundary Enhancement Loss: This component penalizes misclassifications at the boundaries between adjacent glomeruli, ensuring clear separation.

$$\mathcal{L}_{\text{boundary}}=\frac{1}{N_{\text{boundary}}}\sum_{j\in\text{boundary}} \left\| p_{j}-y_{j} \right\|^{2}$$

where $N_{\text{boundary}}$ is the number of boundary pixels, $p_{j}$ and $y_{j}$ are the predicted and ground truth values for boundary pixel $j$, $\lambda$ is a weighting factor that balances the contribution of the boundary loss relative to the region loss.

The total loss function is defined as:

$$L_{\text{total}}=L_{\text{seg}}+\lambda L_{\text{boundary}}$$

where $L_{\text{seg}}$ is the region segmentation loss, $L_{\text{boundary}}$ is the boundary enhancement loss, and $\lambda$ is a weighting factor to balance the two components.

This adaptive loss function ensures that the model pays special attention to both the overall segmentation accuracy and the challenging boundary regions, significantly improving its performance in densely packed glomerular structures.

**Fine-Tuning Approach** To further enhance the model's ability to handle complex cases, we implemented a progressive fine-tuning strategy with the following key steps:

Learning Rate Scheduling:

We initialized the fine-tuning process with a reduced learning rate ($lr_{\text{initial}}=1\times{10}^{-5}$) to allow for gradual and stable updates to the model weights. A cosine annealing scheduler was employed to dynamically adjust the learning rate during training, ensuring optimal convergence:

$$lr(t)=lr_{\text{min}}+\frac{1}{2}(lr_{\text{max}}-lr_{\text{min}})\left( 1+cos\left( \frac{t}{T}\pi\right) \right)$$

where $lr_{\text{min}}$ and $lr_{\text{max}}$ are the minimum and maximum learning rates, $t$ is the current training step, $T$ is the total number of steps.

Data Augmentation for Fine-Tuning:

During fine-tuning, we applied targeted data augmentation techniques, including random rotation, scaling, and elastic deformations, to simulate variations in glomerular morphology and spatial arrangements. Special emphasis was placed on augmenting regions with closely adjacent glomeruli to improve the model's robustness in challenging scenarios. The detailed workflow was shown in **Supplemental Figure 10**.

**Strategies to address data imbalances**

**Data-Level Strategies:** Oversampling of Minority Class: The number of globally sclerotic glomeruli was increased by four times through oversampling using augmentation techniques, including random rotation, flipping, and elastic deformations.

Augmentation Techniques: Each globally sclerotic glomerulus was augmented using random rotation (±30°), horizontal and vertical flipping, and elastic deformations to simulate tissue variability.

**Algorithm-Level Strategies:** Weighted Loss Function: A weighted cross-entropy loss was applied to address class imbalance, assigning higher weights to the minority class and lower weights to the majority class. The weighted loss function is defined as:

$$L_{\text{weighted}}=-\frac{1}{N}\sum_{i=1}^{N} \left( w_{\text{minority}}y_{i}log(p_{i})+w_{\text{majority}}(1-y_{i})log(1-p_{i}) \right)$$

where $w_{\text{minority}}$ and $w_{\text{majority}}$ are the class weights.

To focus on hard-to-classify samples, focal loss was incorporated. The focal loss is defined as:

$$L_{\text{focal}}=-\alpha(1-p_{t})^{\gamma}log(p_{t})$$

where $\alpha$ is a balancing factor, $p_{t}$ is the predicted probability for the true class, and $\gamma$ is the focusing parameter.

**Clinical and pathological data collection**

The clinical and laboratory data at the time of kidney biopsy were obtained retrospectively from the medical records including age, gender, BMI (body mass index), blood pressure, 24-h proteinuria, serum creatinine ,eGFR (according to the Chronic Kidney Disease Epidemiology Collaboration [CKD-EPI] equation)^1^. All the kidney biopsy specimens were stained with Masson’s trichrome, periodic acid–Schiff, hematoxylin and eosin, and silver with standard protocols. The biopsies were evaluated under the light microscopy outlined in the MEST-C criteria in Oxford classification for IgA nephropathy. Mesangial hypercellularity (M1) was defined as an M score of ≧0.5; Endocapillary hypercellularity (E) and segmental sclerosis (S) were scored as absent (0) or present (1). Interstitial fibrosis/tubular atrophy (T) was scored according to the estimated percentage of interstitial fibrosis and tubular atrophy in the cortex: T0 (≤25% of the cortex), T1 (26%–50%), and T2 (>50%). Crescents (C) were scored according to the fractions of glomeruli with cellular or fibrocellular crescents: C0 (no crescents), C1 (<25%), and C2 (≥25%).

**Supplemental Figure 1. The architecture of the ResNet50 training framework.** The framework comprises data preprocessing, a ResNet50-based backbone for feature extraction, and a head module for classification. In the data preprocessing stage, glomeruli of 1000x1000 size are extracted from the source image, followed by oversampling to balance the dataset. Data augmentation techniques are then applied to enhance model generalization. The backbone network adopts ResNet50, where the input image is first processed through an initial layer before passing through multiple residual blocks. Each ResBlock consists of a downsampling operation and residual connections to facilitate gradient flow and improve feature learning. After passing through three stacked residual blocks, the extracted features are forwarded to the head module, where a multilayer perceptron (MLP) processes them for classification. The final classification output is supervised by a hybrid loss function, comprising $\mathcal{L}_{local}$ and $\mathcal{L}_{weighted}$, which are computed based on the ground-truth labels. These loss functions help to refine the model's ability to distinguish between globally sclerotic glomeruli (GS) and non-GS glomeruli, ensuring accurate classification and robust model performance.

**Supplemental Figure 2.** **Non-linear association between quantitative glomerular characteristics and ESKD or ≥50% reduction in eGFR.** Association of quantitative glomerular morphological characteristics and adjusted HR of 50% eGFR decline or ESKD in patients with IgAN. Three knots were used to model restricted cubic splines. The estimated risk ratio is depicted by the solid line, while the 95% CI is represented by the shaded area. Models were adjusted for age, gender, and BMI.

**Supplemental Figure 3.** **The association between glomerular morphological characteristics and ESKD or ≥30% reduction in eGFR.**

**Supplemental Figure 4.** **Kaplan-Meier survival curves based on quantitative glomerular characteristics in patients with eGFR <30 ml/min/1.73m^2^.** Optimal cut-offs for all glomerular morphological characteristics were determined by computing the maximally selected log-rank statistic using the “survminer” R package.

**Supplemental Figure 5. Kaplan-Meier survival curves based on quantitative glomerular characteristics in patients with eGFR ≥60 ml/min/1.73m^2^.** Optimal cut-offs for all glomerular morphological characteristics were determined by computing the maximally selected log-rank statistic using the “survminer” R package.

**Supplemental Figure 6. Kaplan-Meier survival curves based on quantitative glomerular characteristics in patients with 30≤eGFR <60 ml/min/1.73m^2^.** Optimal cut-offs for all glomerular morphological characteristics were determined by computing the maximally selected log-rank statistic using the “survminer” R package.

**Supplemental Figure 7.** **Non-linear association between quantitative glomerular characteristics and ESKD or ≥30% reduction in eGFR.** Association of quantitative glomerular morphological characteristics and adjusted HR of 30% eGFR decline or ESKD in patients with IgAN. Three knots were used to model restricted cubic splines. The estimated risk ratio is depicted by the solid line, while the 95% CI is represented by the shaded area. Models were adjusted for age, gender, and BMI.

**Supplemental Figure 8.** **Non-linear association between quantitative glomerular characteristics and ESKD or ≥30% reduction in eGFR based on four knots.** Association of quantitative glomerular morphological characteristics and adjusted HR of 30% eGFR decline or ESKD in patients with IgAN. Four knots were used to model restricted cubic splines. The estimated risk ratio is depicted by the solid line, while the 95% CI is represented by the shaded area. Models were adjusted for age, gender, and BMI.

**Supplemental Figure 9.** **Non-linear association between quantitative glomerular characteristics and ESKD or ≥30% reduction in eGFR based on five knots.** Association of quantitative glomerular morphological characteristics and adjusted HR of 30% eGFR decline or ESKD in patients with IgAN. Five knots were used to model restricted cubic splines. The estimated risk ratio is depicted by the solid line, while the 95% CI is represented by the shaded area. Models were adjusted for age, gender, and BMI.

**Supplemental Figure 10.** **The architecture of the Mask R-CNN training framework.** The framework consists of multiple stages, including data preprocessing, backbone feature extraction, region proposal generation, fine-tuning, and post-processing. In the data preprocessing stage, the source image is cropped into 4000x4000 patches with a 20% overlap rate, followed by a dual-threshold filtering mechanism to refine the patches before feeding them into the model. The backbone utilizes a Swin Transformer to extract hierarchical feature maps, which are then processed by the neck module. The neck consists of a Region Proposal Network (RPN) that generates candidate regions of interest (ROIs), further refined through a convolutional neural network (CNN) to improve proposal quality. In the head module, the selected ROIs are passed through a multilayer perceptron (MLP) to generate mask predictions. The model is optimized using a hybrid loss function consisting of $\mathcal{L}_{region}$ and $\mathcal{L}_{boundary}$, which enhance segmentation accuracy by leveraging both regional and boundary information. To further improve performance, fine-tuning strategies such as a cosine annealing learning rate scheduler and data augmentation techniques are applied during training. Finally, in the post-processing stage, the predicted masks undergo refinement using a non-maximum suppression algorithm to eliminate redundant detections and enhance final segmentation quality.

**Supplemental Table 1. Calculation of morphological characteristics of glomeruli**

| **Characteristic** | **Description** |
| --- | --- |
| Area | Area of the segmented structure (μm^2^) |
| Diameter | Diameter of the largest circle fully fitting inside the structure (μm) |
| Perimeter | The total length around the outer edge of the glomerular capsule (μm) |
| Elongation | Function of the length of the structure's minor and major axis. The elongation of a circle is 0 and gets higher the more elongated the structure is.  Elongation=$1-\frac{minor\_axis\_length}{major\_axis\_length}$ |
| Eccentricity | Ration of the distance between the structure's focal points over the major axis' length. The eccentricity equals 0 for a circle and 1 for ellipses.  Eccentricity=$\frac{\sqrt{{major\_axis\_length}^{2}-{minor\_axis\_length}^{2}}}{major\_axis\_length}$ |
| The percentage of sclerotic glomeruli | The percentage of sclerotic glomeruli relative to the total number of glomeruli in a single whole slide image (WSI). |

Note: The description provided above primarily refers to the work of Hölscher et al.^2^

**Supplemental Table 2. Ablation study of optimization strategies for glomerular segmentation model**

| **Model** | **Dice** | **Jaccard** | **ASD** | **F1-score** |
| --- | --- | --- | --- | --- |
| Baseline | 0.8 | 0.72 | 42.35 | 0.8 |
| + R.S. Loss | 0.84 | 0.76 | 36.2 | 0.84 |
| + B.E. Loss | 0.87 | 0.79 | 31.45 | 0.87 |
| **Ours** | **0.91** | **0.83** | **28.81** | **0.91** |

**Supplemental Table 3. Ablation study of optimization strategies for glomerular classification model**

| **Model** | **Recall** | **Precision** | **Accuracy** | **F1-score** | **AUC** |
| --- | --- | --- | --- | --- | --- |
| Baseline | 0.87 | 0.85 | 0.88 | 0.86 | 0.84 |
| + Data-Level | 0.91 | 0.89 | 0.92 | 0.9 | 0.88 |
| + Algorithm-Level | 0.93 | 0.92 | 0.94 | 0.93 | 0.91 |
| **Ours** | **0.96** | **0.97** | **0.97** | **0.965** | **0.94** |

**Supplemental Table 4. The association between glomerular morphological characteristics and second endpoint in patients with IgAN**

| Characteristics | Model 1 | | |  | Model 2 | | |  | Model 3 | | |
| --- | --- | --- | --- | --- | --- | --- | --- | --- | --- | --- | --- |
|  | HR | 95% CI | P |  | HR | 95% CI | P |  | HR | 95% CI | P |
| glomerular area ≤ 16726.33 μm2 | 1.6 | 1.15-2.23 | 5.00×10^-3^ |  | 1.52 | 1.08-2.15 | 0.02 |  | 1.31 | 0.92-1.86 | 0.13 |
| glomerular diameter ≤ 157.87 μm | 1.6 | 1.15-2.23 | 6.00×10^-3^ |  | 1.51 | 1.07-2.14 | 0.02 |  | 1.3 | 0.92-1.85 | 0.14 |
| glomerular perimeter ≤ 457.01 μm | 1.66 | 1.19-2.31 | 3.00×10^-3^ |  | 1.57 | 1.11-2.23 | 0.01 |  | 1.35 | 0.95-1.92 | 0.1 |
| glomerular elongation ≤ 0.1255 | 1.5 | 1.00-2.25 | 0.05 |  | 1.28 | 0.84-1.95 | 0.26 |  | 1.51 | 0.98-2.32 | 0.06 |
| glomerular eccentricity ≤ 0.4623 | 1.7 | 1.14-2.53 | 9.00×10^-3^ |  | 1.42 | 0.94-2.17 | 0.1 |  | 1.7 | 1.11-2.60 | 0.01 |
| GS percentage ≥ 44.23% | 4.48 | 3.12-6.41 | 2.99×10^-16^ |  | 3.48 | 2.27-5.31 | 8.43×10^-9^ |  | 2.44 | 1.56-3.81 | 9.60×10^-5^ |

Note: Model 1: unadjusted; Model 2: Adjusted for age, gender, BMI, baseline proteinuria, eGFR, and MAP at kidney biopsy. Model 3: Model 2 and MEST-C. The second endpoint was ESKD or a ≥30% decline in eGFR.

**Supplemental Table 5. Benchmarking results of candidate models across segmentation and classification metrics**

| **Segmentation Models** | | | | **Classification Models** | | | | |
| --- | --- | --- | --- | --- | --- | --- | --- | --- |
| **Model** | **Dice** | **Jaccard** | **ASD** | **Model** | **Recall** | **Precision** | **Accuracy** | **AUC** |
| YOLACT++ | 0.76 | 0.68 | 48.20 | EfficientNet | 0.85 | 0.83 | 0.86 | 0.81 |
| SOLOv2 | 0.78 | 0.70 | 45.80 | VGG16 | 0.82 | 0.80 | 0.83 | 0.79 |
| **Mask R-CNN** | **0.80** | **0.72** | **42.35** | **ResNet50** | **0.87** | **0.85** | **0.88** | **0.84** |

**References**

1. Levey AS, Stevens LA, Schmid CH, et al. A new equation to estimate glomerular filtration rate. *Annals of internal medicine* 2009;150(9):604-12. doi: 10.7326/0003-4819-150-9-200905050-00006 [published Online First: 2009/05/06]

2. Hölscher DL, Bouteldja N, Joodaki M, et al. Next-Generation Morphometry for pathomics-data mining in histopathology. *Nature communications* 2023;14(1):470. doi: 10.1038/s41467-023-36173-0 [published Online First: 2023/01/29]
